# Rethinking the interplay between affluence and vulnerability to aid climate change adaptive capacity

**DOI:** 10.1007/s10584-020-02819-x

**Published:** 2020-10-26

**Authors:** Christine Eriksen, Gregory L. Simon, Florian Roth, Shefali Juneja Lakhina, Ben Wisner, Carolina Adler, Frank Thomalla, Anna Scolobig, Kate Brady, Michael Bründl, Florian Neisser, Maree Grenfell, Linda Maduz, Timothy Prior

**Affiliations:** 1 Center for Security Studies, Swiss Federal Institute of Technology (ETH), Zürich, Switzerland; 2 Australian Centre for Culture, Environment, Society and Space (ACCESS), School of Geography and Sustainable Communities, University of Wollongong, Wollongong, Australia; 3 Department of Geography and Environmental Sciences, University of Colorado Denver, Denver, CO USA; 4 Fraunhofer Institute for Systems and Innovation Research ISI, Karlsruhe, Germany; 5 Institute for Risk and Disaster Reduction, University College London, London, UK; 6 Environmental Studies Department, Oberlin College, Oberlin, OH USA; 7 Mountain Research Initiative, University of Bern, Bern, Switzerland; 8 Climate and Disaster Risk Research and Consulting, Sydney, Australia; 9 Environmental Governance and Territorial Development Institute, University of Geneva, Geneva, Switzerland; 10 Australian Red Cross, North Melbourne, VIC Australia; 11 University of Melbourne, Melbourne, VIC Australia; 12 WSL Institute for Snow and Avalanche Research SLF, Davos Dorf, Switzerland; 13 Fraunhofer Institute for Technological Trend Analysis INT, Euskirchen, Germany; 14 Resilient Melbourne, Melbourne, VIC Australia; 15 Control Centre, IT, Network & Infrastructure, Swisscom (Schweiz) AG, Ittigen, Switzerland; 16 Wonder Labs, San Jose, CA USA

**Keywords:** Climate change adaptation, Disaster resilience, Natural hazards, Psychosocial coping capacity, Social vulnerability

## Abstract

Affluence and vulnerability are often seen as opposite sides of a coin—with affluence generally understood as reducing forms of vulnerability through increased resilience and adaptive capacity. However, in the context of climate change and an increase in associated hazards and disasters, we suggest the need to re-examine this dynamic relationship—a complex association we define here as the Affluence–Vulnerability Interface (AVI). We review research in different national contexts to show how a more nuanced understanding of the AVI can (a) problematize the notion that increasing material affluence necessarily has a mitigating influence on social vulnerability, (b) extend our analysis of social vulnerability beyond low-income regions to include affluent contexts and (c) improve our understanding of how psychosocial characteristics influence people’s vulnerability. Finally, we briefly outline three methodological approaches that we believe will assist future engagement with the AVI.

## Introduction

Despite economic and technological progress, floods, wildfires, drought and other natural hazards continue to impact communities in acute and prolonged ways around the world (Bouwer 2011; Hallegatte 2013; Mechler and Bouwer 2015; Thomalla et al. 2006). Exacerbated by climate change, a range of hazards are projected to become more frequent and intense (IPCC 2014; Hoegh-Guldberg et al. 2018). The social and economic consequences of these climate-exacerbated hazards are likely to remain significant (Bouwer 2011; Eriksen and Ballard 2020; Kinoshita et al. 2016; Lucas et al. 2020; IPSP 2018).

In this evolving context, three related issues have become important to address. First, general material affluence (marked by an increase in per capita GDP and technological advancement) is not enough to mitigate climate change or to cope with the impacts of associated natural hazards, especially for people who are socially and/or physically marginalized (Fothergill and Peek 2004; Kelman 2015; Cinner et al. 2018). A problem of growing inequity is evident in all countries—high, medium and low income, especially in the context of rapid urban development processes. In some cases, increasing material affluence and access to resources may actually exacerbate pre-existing social vulnerabilities and produce new risks and vulnerabilities (Beck 1992; Simon 2014; Eadie and Su 2018). Even well-developed infrastructure, which we associate with technological advancement, can lead to segregation and inequality (Graham and Marvin 2001). Second, analysis of social vulnerability should extend beyond marginal and less economically developed regions to include acute forms of social precariousness and persistent climate change-related vulnerabilities in affluent settings. Third, a focus on material, financial and institutional resources has rendered important psychosocial characteristics underemphasized. Individual and community social and psychological attributes must also be acknowledged as influential, if not overriding, factors that influence social vulnerability to hazards in the face of climate change.

In the past decades, disaster risk reduction (DRR) policy has evolved from a sole emphasis on the mechanical and structural aspects of hazard mitigation to the guiding principle of disaster resilience. Resilience puts a strong emphasis on distributed capacities for disaster prevention, preparedness and recovery, and explicitly recognizes the need for social vulnerability analysis (Wisner et al. 2004; Pelling 2007; Tierney 2014; UN 2015; UNDRR 2015; Oliver-Smith et al. 2017). Nevertheless, challenges remain in how we engage in meaningful ways with historical, subjective and relational aspects of social vulnerability. Our essay speaks to this gap by providing theoretical insights and methodological pathways from research experiences in different national contexts. A particular focus on the USA, Switzerland and Australia demonstrates that affluent societies or places often have considerable and rising levels of inequality and it is the relative deprivation and marginalization of individuals and communities within these societies rather than affluence on the aggregate level, which result in vulnerable populations being hidden, forgotten or created.

In this paper, we draw on the concept of the Affluence–Vulnerability Interface (AVI), developed by Eriksen and Simon 2017, to examine these points with respect to individuals’ and communities’ adaptive capacity to climate change and associated natural hazards and disasters. We argue that the AVI is multifaceted and that this complexity should be more directly acknowledged in climate change and hazards research. The use of the AVI abbreviation should not be interpreted as an attempt to diminish these complexities. This focus enables us to (a) better understand why, in many contexts, the impacts of natural hazards remain high (Munich 2019) despite a general increase in global material wealth (Lange et al. 2018) and (b) suggest effective research and policy pathways that will help governments and emergency practitioners promote greater adaptive capacity to climate change-related hazards.

## Vulnerability and affluence in current DRR research and practice

According to Adger and Kelly (1999, 256), ‘the extent to which individuals, groups or communities are ‘entitled’ to make use of resources determines the ability of that particular population to cope with and adapt to stress’. This perspective suggests that peoples’ abilities to access and utilize resources will mediate adaptation to climatic changes and resulting environmental and social stressors. Yet, what exactly is meant by the term ‘resources’ (Abramson et al. 2010; Hobfoll 2012)? In recent research, material wealth is one common factor associated with peoples’ capacities to cope with climate-exacerbated natural hazards (Fothergill and Peek 2004; Hallegatte 2013; Klomp and Valckx 2014; Mechler and Bouwer 2015; Schumacher and Strobl 2011). This view typically assumes a direct link between increased affluence (and associated access to material resources) and improved adaptive capacity. In other words, if we manage to increase the material wealth of an individual, community or nation, an increase in resilience will necessarily follow due to the availability of, for example, advanced infrastructure, technology and social services. In the following sections, we unpack and complicate this assumed linear relationship.

### An ongoing fixation with material coping capacities

As an important element of risk, understanding what and who is vulnerable, and where vulnerable assets or people are located, has become a central aspect of DRR policies and research (Cutter and Finch 2008; Hearn Morrow 1999). Today, there is broad empirical evidence showing that the social characteristics of people and communities—including their social marginalization—have an indisputable influence on the severity of a disaster (Collins 2010; Haque and Etkin 2007; Ismail-Zadeh et al. 2017; Quarantelli 1992; Sword-Daniels et al. 2018; Wisner 1998). To some extent, this knowledge has been reflected in DRR policy documents and international strategies since the release of the Hyogo Framework for Action 2005–2015 (UNDRR 2005). These insights have led to important policy developments, such as the Sendai Framework for Disaster Risk Reduction (2015–2030), which place a strong emphasis on understanding and mitigating the underlying drivers of disaster risk (UNDRR 2015). Material disadvantages and vulnerability (of housing and critical infrastructure, for instance) are likewise considered to increase disaster risk in documents outlining climate change adaption strategies (UN 2015; UNFCCC 2015).

Following this perspective, the United Nations Office for Disaster Risk Reduction (UNDRR) defines social vulnerability as those ‘characteristics determined by physical, social, economic and environmental factors or processes which increase the susceptibility of an individual, a community, assets or systems to the impacts of hazards’ (UNDRR 2017). The Intergovernmental Panel on Climate Change (IPCC) positions social vulnerability as a ‘determinant of risk’, defining it as the ‘susceptibility, sensitivity, and lack of resilience or capacities of [an] exposed system to cope with and adapt to extremes and non-extremes’ (Cardona et al. 2012, 71). Thus, social vulnerability reflects that character of a place or population that limits its ability to cope with, or adapt to, disturbance, change or stress (Rumbach et al. 2016).

Although our understanding of social vulnerability in climate change and disaster contexts has evolved substantially from early assessments (Füssel and Klein 2006), a wider uptake of this knowledge has been slow. Castree (2017) and Haque and Etkin (2007, 271) constructively critique some members of the physical science community, as well as public sector decision-makers who ‘have not yet accepted the idea that understanding and using human and societal dimensions is equally or more important than trying to deal with and control nature through the use of technology’. There are four core reasons for this deficiency.

First, while social vulnerability is increasingly considered in climate change policy, most applied and region-specific management activities focus on emergency response and short-term recovery. This is at the expense of targeted investment in long-term mitigation measures, which has been shown to both reduce social vulnerability to associated disasters and alleviate poverty (de Vet et al. 2019). Broadly speaking, economic growth is considered mainly in light of its mitigative influence on vulnerability, even though it can also generate social vulnerability and inequity (Eadie and Su 2018). This undervalues the role of development as a driver of risk (Thomalla et al. 2018). It also misses opportunities to examine how climate change affects health inequalities linked to access and distribution, which are distorted by affluence. This ranges from mortality from extreme events to food insecurity and access to public goods (Campbell et al. 2016; Myers et al. 2017). For example, Watts et al. (2015) argue that tackling climate change could be the greatest global health opportunity of the twenty-first century.

Second, a focus on strengthening material capacities for disaster response and recovery tends to overshadow the need to understand people’s everyday lived experiences of social and physical vulnerabilities (Tanner et al. 2015). This trend is reinforced by various attempts to index and compare the vulnerability of communities, regions or whole nations in a fashion that fails to illuminate less obvious expressions of social vulnerability. Applied research tends to establish broad-scale vulnerability assessment strategies (regional or national scale), which are not fine-grained enough to assess heterogeneous households, diverse social conditions and individual characteristics. These widely circulated awareness-raising documents, such as reports by the IPCC (2014) and the Social Vulnerability Index (ATSDR 2017), lack historical, place-based assessments of policies, plans and decisions that are often complicit in the production of social vulnerabilities.

Third, a long tradition of social science scholarship that actually addresses the first two points (Wisner 1993; Wisner et al. 2004; Hewitt 1997; Liverman 1990) remains largely unknown to many climate change adaptation managers in public, private and non-profit sectors due to disciplinary divides and the inaccessibility of some academic writing styles, together with the unaffordability of many academic publications. These obstacles prevent the central points of this rich strand of research from being taken up by practitioners.

Fourth, understanding what actually causes social vulnerability in diverse contexts is extremely challenging. Social and psychological characteristics of individual people, communities and places are multifaceted, methodologically difficult to access and costly to measure. For example, individuals and communities may have excellent coping capacities with respect to some climate change impacts while simultaneously being vulnerable to others. It is difficult to incorporate these complex elements into the often synoptic nature of current social vulnerability analyses (Brown and Westaway 2011).

### The undervalued role of non-material coping capacities

Affluence is typically understood as the ability of an individual or community to achieve a financial status that enables a corresponding level of access to material resources and/or assets (Adger 2006; Adger and Kelly 1999; Smit and Wandel 2006). Increased material affluence—particularly growing wealth and technological advancement—represents the social change that is most often associated with reduced hazard vulnerability, increased resilience, and improved adaptation and coping capacity (Mcleod and Kessler 1990; Thomalla et al. 2006). To be sure, this is often the case, as these material resources can (at least theoretically) be easily converted into risk-mitigating adaptations, such as buying a car that enables a family to evacuate from a hurricane or building an embankment to mitigate against sea-level rise. The experience of unequal risk during Hurricane Katrina in 2005 highlighted how affluent people and places in New Orleans, Louisiana, USA, were better able to cope before, during and after the hurricane (Cutter and Emrich 2006; Fothergill and Peek 2015).

Yet, a growing body of robust research examining the formation of social vulnerability demonstrates that a focus on material wealth alone does not always adequately capture the interplay between climate-exacerbated natural hazards, social vulnerability and loss (Aldrich 2012; Barnett et al. 2016; Collins 2010; Collins and Bolin 2009; Eriksen and Simon 2017; Kuhlicke et al. 2011; Roth et al. 2018; Haworth et al. 2019). In taking a more cautious approach when describing trends in social vulnerability, it is possible to demonstrate that climate change-related risks and vulnerabilities are not improving proportional to levels of economic development (Eriksen 2019a).

Resources influencing the coping capacity of communities and individuals related to natural hazards can be material (e.g. finances, assets, technologies), psychosocial (e.g. emotions, social networks, belonging, social cohesion, beliefs, local knowledge) and institutional (e.g. political power, governance functionality) (Hobfoll 2012; Simon 2014; Sword-Daniels et al. 2018; Lakhina et al. 2019). Material and non-material resources—also referred to as ‘tangible’ and ‘intangible’ (Tapsell et al. 2002; de Andrade and Szlafsztein 2018)—may interact to influence institutional resources and social vulnerabilities. Meanwhile, the existence, capacity and accountability of government institutions (local to national) have a strong influence on coping capacity. We use the term ‘coping capacity’ here to reference the ability of people, organizations and systems, using available skills and resources, to manage adverse conditions, risk or disasters (Gaillard 2010).

We argue below that expanding our understanding of the relationship between affluence and social vulnerability is important and suggest that this association is not linear. Rather, the relationship must be understood as dynamic and complex in the evolving context of climate change and its increasingly frequent and severe consequences for communities worldwide.

## The Affluence–Vulnerability Interface: three critical insights and proposed research directions

With the objective of generating a more robust and comprehensive understanding of the AVI across a range of contexts and locations, we now reflect on insights from a number of independent research projects from different national contexts. The AVI was initially developed as a process-based analytical framework to unpack the concomitant yet competing social conditions that shaped short- and long-term experiences of wildfire recovery in California, USA. The study results demonstrated that:

Explicitly integrating and examining the role of, for example, affluence and privilege, risk and vulnerability, age and disability within a single study, instead of just highlighting differences and disparities … recognizes class and affluence as a financial status that intersects with other cultural practices, identities and politics, which are based on ideological norms and are lived but often generalized and/or unacknowledged. (Eriksen and Simon 2017, 296–297)

We expand on these insights to demonstrate the broader analytic and methodological utility (summarized in Table 1) of the AVI in (a) problematizing the frequently assumed linear and mitigative relationship between affluence and social vulnerability (Section 3.1), (b) extending analysis of social vulnerability to include acute climate change and related hazard vulnerabilities in affluent settings (Section 3.2) and (c) improving our understanding of the vital role of psychosocial coping capacity in formulating socially responsive DRR policy and practice (Section 3.3).

**Table 1:** Analytical approaches recommended to investigate the Affluence–Vulnerability Interface (AVI), adaptive capacity and climate change

	Analytic approaches		
	Historical-structural	Intersectional	Psychosocial
Aims	~ Identify historical and structural drivers influencing the co-production of affluence and vulnerability ~ Systematically examine and connect past, present and future development processes	~ Locate experiences of vulnerability amidst affluence ~ Generate insights into concomitantly held experiences, conditions and identities	~ Understand people’s coping capacities by examining how psychological factors and the surrounding community context (beyond material wealth) influence well-being and ability to function
Scales of analysis	~ Often begins with the policy realm and extends to policy implementation, impacts and outcomes	~ Often begins with societal or community scales and extends to households and individuals	~ Often begins with individuals and households and extends to neighbourhood and community scales
Insights from AVI analysis of factors that increase or reduce vulnerability to climate-exacerbated hazards and disasters	~ Urban development with significant financial benefits for some simultaneously produces considerable risks for others, as homes and lives are put in harm’s way when cities expand into more hazard-prone areas ~ Diverse vulnerabilities unfold as part of a region’s broader historical development, which may feed into persistent social injustices, inequalities and vulnerabilities that are reproduced during disaster recovery and rebuilding processes	~ Pockets of marginalization within affluent communities, such as undocumented/ irregular/ seasonal migrants who may be unacknowledged in climate change mitigation and adaptation efforts ~ Affluent individuals or households who may be socially disadvantaged due to conditions, such as age, limited mobility, poor health or racism, may not derive any benefits from their otherwise secure financial position ~ Everyday (‘mundane’) vulnerabilities that exist in ‘plain sight’, such as language barriers, access or functional needs, are often overlooked by official disaster mitigation efforts	~ Social cohesion can aid the development of mitigative behaviour and increase psychosocial coping capacities, for example, through the work of mutual aid groups, voluntary networks and neighbourhood associations ~ Marginalization and discrimination underpinned by social characteristics are often magnified in disasters, undermining people’s ability to cope ~ When marginalized voices are acknowledged and included in emergency management processes, communities and institutions often benefit from the diverse forms of embodied and embedded knowledge

Source: authors

### AVI insight 1: the co-production of affluence and social vulnerability and the merits of historical-structural analysis

Focusing on the relationship between affluence and social vulnerability entails systematically examining how development processes, which enable cities and regions to thrive and become wealthier, may also increase individual and collective vulnerability to climate change and natural hazards. This perspective shows how affluence can play a central role in the creation of both disaster resilience and vulnerability. Here, we briefly highlight the latter. It is clear that increased material wealth will in many cases lessen conditions of social vulnerability. Yet, if we understand affluence to have only a mitigating influence, then we are likely to miss circumstances where vulnerabilities and forms of social risk exist as a consequence of policies aimed at increasing economic growth and material wealth (Beck 1992).

Historical analysis of the lucrativeness of suburban landscapes provides an insightful example of how affluence and social vulnerability to climate-exacerbated hazards, such as wildfires and floods, are co-produced (Simon 2014; Collins 2010; Kaufman 2017). Suburban landscapes in the Western USA simultaneously produce and maintain financial benefits, risk and vulnerability (Simon 2017). The pursuit of affluent neighbourhoods and construction industry profits at the city’s edge in the USA (and elsewhere) are not benign. Over time, the generation of material benefits have coincided with the production and maintenance of considerable risks related to wildfires, as homes and lives are put in harm’s way with the expansion of cities into historically fire-prone areas (Kramer et al. 2018). This includes low-income trailer parks, positioned in high-risk locations, for those who cannot afford more expensive city homes (Rumbach et al. 2020).

Such historical insights illuminate how social vulnerability is generated within landscapes that are intentionally altered, developed and maintained in a manner that retains their productivity, or desired economic purpose, for cities (property tax revenue), developers (construction contracts) and landholders (subdivide-and-sell) alike. Social vulnerability is much more than simply a produced outcome or material inscription (Wisner et al. 2004; Oliver-Smith et al. 2017). Employing a historical-structural research agenda reveals how diverse vulnerabilities unfold as part of a region’s broader historical development, which feed into persistent social injustice, inequality and vulnerability. When historically minded research is incorporated into one’s practice (e.g. by using recent and archived land use planning and economic development documents), it is easier to highlight persistent root causes of social vulnerability. As with all analysis of complex systems, there are caveats attached to this approach, which require careful attention. Historical-structural analysis can lead to the discovery of secondary and tertiary cascading vulnerability impacts within other populations and locations. Further research is required to probe these ‘knock on effects’ of change, which are associated with the coproduction of affluence and risk.

### AVI insight 2: locating vulnerability amidst affluence and the benefits of intersectional analysis

Expanding our understanding of the relationship between affluence and social vulnerability also entails exploring sites of acute vulnerability within affluent areas. Rich empirical evidence suggests that social vulnerability is a significant root cause of most disasters (see Roth et al. 2017 for an overview). However, social vulnerability to natural hazards is highly context specific. Of particular relevance here are pockets of disadvantaged groups susceptible to climate change, who live alongside less vulnerable groups in highly affluent communities (Eriksen and Simon 2017; Roth et al. 2018; Simon 2014; Wisner 1998; Lakhina et al. 2019; Maidl and Buchecker 2015).

For example, the city of Zürich in Switzerland is consistently ranked as one of the most liveable and wealthy on the planet. Yet, it is also home to a growing number of socially vulnerable people who live in informal and/or precarious housing (Prior et al. 2017; Roth et al. 2018). Factors that influence social vulnerability include the following: (a) people living in social housing areas close to flood zones; (b) non-German-speaking residents unable to understand risk information provided by the authorities in German, or who are unfamiliar with Switzerland’s system of hazard-specific siren warnings; (c) the elderly; and (d) *sans papiers* migrants (undocumented status) who work for cash but are not registered in the city and are therefore unacknowledged in climate change mitigation and adaptation efforts. Social workers highlighted that people socially vulnerable in their everyday lives (because of mobility or health issues, isolation, mental illness, low income, etc.) were disproportionately more likely to be impacted by natural hazards, in part because ‘mundane’ vulnerabilities that exist in ‘plain sight’ often are overlooked by official disaster mitigation efforts (Roth et al. 2018).

On the one hand, these overlooked vulnerabilities may point to pockets of marginalization within affluent communities (e.g. the *sans papiers* in Zürich). On the other hand, these vulnerabilities may arise when a relatively safe (perhaps even affluent) individual or household is disadvantaged socially due to conditions that undercut any benefits derived from their otherwise secure financial position. Examples might include the non-ambulatory residents, the elderly or young children who have difficulty evacuating; people suffering from mental health issues who may find it difficult to recover from, or cope with, the aftermath of a disaster; or people of colour who face discrimination within the vast legal and insurance landscape confronting disaster survivors and displaced people (Astill and Miller 2016; Farbotko and Lazrus 2012; Fothergill and Peek 2015; Lister 2014; Simon 2017).

In light of these social complexities, we suggest that researchers and emergency managers alike utilize intersectional climate hazards analysis (Kaijser and Kronsell 2014; Walker et al. 2019). Intersectional analysis can provide a useful framework for generating insights into concomitantly held experiences, conditions and identities. For example, it can highlight how expressions of affluence and social vulnerability overlay or intersect one another, thereby illuminating and disentangling the complicated social fabric confronting climate change and DRR efforts (Eriksen and Simon 2017). There is a need for further research that undertakes nuanced analysis of affluent contexts in order to tease out particular cultural and historical manifestations of social vulnerability. This includes a focus on the manifestation of differing levels of affluence and vulnerability experienced by different strata of the rapidly expanding middle class in most countries of the world (e.g. see Zuniga and Campos 2013).

### AVI insight 3: understanding social vulnerability through analysis of psychosocial coping capacity

A third approach to examining the AVI involves broadening our assessment of what social factors influence individual hazard vulnerabilities. Specifically, we believe that it is important to acknowledge not only material coping capacities (which privilege the influence of affluence) but also psychosocial coping capacities, which shape the acute and chronic social impacts of disasters (Tapsell 2010). We consider psychosocial coping capacity to be the combined influence of psychological factors and the surrounding community context on an individual’s physical and mental wellness and their ability to function (Eyre 2017).

Recent studies demonstrate different dimensions of psychosocial coping capacity and their influence on social vulnerability and adaptation to climate-related hazards. On the one hand, socio-cultural narratives can create conflicting perceptions of, and responses to, climate change, which legitimize unequal resource distribution and justify the suppression or capitalisation of both sub-cultural and individual risk perceptions (Rühlemann and Jordan 2019). On the other hand, social cohesion, particularly characteristics like ‘sense of community’ and ‘collective problem solving’, can act as community-based factors (i.e. social resources) that support the development of mitigative behaviour (i.e. practical resources) and cognitive abilities (i.e. psychological resources). Together, these factors have been shown to increase psychosocial coping capacities among residents at risk of wildfire in Southeast Australia and the US West (Paveglio and Edgeley 2017; Prior and Eriksen 2013). Among disaster recovery workers, research has also shown that factors supporting psychosocial coping capacity, such as the ability to confide, reflect, debate, grow and heal through mental, spiritual and physical safe spaces, are crucial for their continued ability to cope and care amidst social and environmental uncertainty (Eriksen 2019b; see also Brady 2011). These same factors increase psychosocial coping capacities and overall well-being in communities with greater economic equality and high social cohesion, suggesting that ‘relative’ well-being is more important than ‘absolute’ wealth in reducing social vulnerabilities (Wilkinson 2002; Pickett and Wilkinson 2015).

Equally critical to any understanding of coping capacity is the matter of marginalization—i.e. the treatment of a person, group or concept as insignificant or peripheral due to social characteristics, such as age, gender, sexuality, ethnicity, race, disability, class or education (Wisner et al. 2004). Marginalization is closely related to discrimination—processes, such as racism, homophobia, sexism and ageism, that undermine psychosocial coping capacity. Social characteristics, marginalization and discrimination intersect in everyday life to make people more or less resilient: The more ways a person is marginalized, the less likely they are to have adequate resources to respond and recover in a disaster (Eriksen 2019a). When disaster strikes, the pressure on socially marginalized groups is therefore often magnified and may inhibit access to available support services (Wisner 2010; Collins 2010; Gorman-Murray et al. 2014; Haworth et al. 2019; Pacoma and Delda 2019; Craig et al. 2019).

Insights from the Northern Territory of Australia demonstrate how the socially marginalized status of Indigenous peoples and their belief systems increases social vulnerability to climate change-related hazards and disasters more broadly (Veland et al. 2013). This is because the perspectives, values and psychosocial coping capacities of these communities tend to be systematically ignored in emergency management and climate change adaptation initiatives. Instead, the results of the ongoing disaster of colonization in Indigenous Nations—poverty, ill health and welfare dependence—are labelled as the primary contributors to hazard vulnerability. When these marginalized voices are acknowledged and incorporated, the resulting emergency management processes have been shown to benefit significantly from the psychosocial coping capacities Indigenous peoples have developed over millennia, often in response to social and environmental uncertainty (Veland et al. 2010; Gaillard et al. 2008; Gaillard 2010; Christianson et al. 2013; Eriksen and Hankins 2014).

Social vulnerability assessments focusing only on material coping capacity are insufficient to explain how people experience and adapt to climate-exacerbated natural hazards and disasters because they do not acknowledge systemic social injustices, such as marginalization and discrimination. In order to plan for, and respond to, threats of the future, knowledge about material coping capacity must be complemented with an understanding of psychosocial coping capacity. Furthermore, given increased human mobility, additional research is required to determine whether, genealogically, locations are essential to ‘sense of community’, as they have been in most places until the late twentieth century. This includes the psychosocial coping capacity of refugees and climate migrants whose numbers are growing and who must periodically seek refuge in unfamiliar places (Farbotko and Lazrus 2012; Lewis and Wisner 1981; Lakhina et al. 2019)?

Understanding such conditions will require greater reliance on qualitative and narrative methods, such as embedded ethnographies, in-depth interviews, participatory mapping and action research, which can explain processes of both social marginalization and social cohesion (Scolobig et al. 2015). These methods also unpack related psychosocial conditions, such as (dis)trust, discrimination, loneliness and despair, which can exist independently of material affluence, yet are significant in the context of peoples’ vulnerability and resilience to climate change.

## Conclusion

In this essay, we have examined the complex and multifaceted relationship between affluence and social vulnerability in the context of climate change and associated hazards and disasters. Our analysis shows that the AVI is a dynamic process by which vulnerability and affluence mutually interact to determine people’s lived experiences of risks and disasters. This suggests that material wealth can have both a mitigative and generative influence on social vulnerability. The AVI is also a condition of spatial difference where acute forms of social vulnerability are nested within and alongside areas of considerable material wealth. They are thus often hidden from view within conventional social vulnerability indices that inform much climate change and DRR policy and practice. Furthermore, the AVI is an area of intentional inquiry that can provide useful insights about individual and community hazard vulnerability while drawing attention to psychosocial coping capacities critical to effective emergency management.

In order to further investigate these aspects of the AVI, and to better respond to future threats, we recommend that the three methodological approaches discussed above and summarized in Table 1—historical-structural analysis, intersectional analysis and psychosocial analysis—are given further attention within research, policy and disaster management settings. We believe that using these approaches to investigate the AVI will augment DRR efforts to improve individuals’ and communities’ adaptive capacity to climate change and associated natural hazards in the years ahead.

Of course, there are many ways material, non-material and institutional resources mediate the relationship between affluence and vulnerability. These dynamics, and the particular forms of social vulnerability they produce, will always be context dependent. Thus, one should not assume that the AVI insights and examples discussed in this essay are perfectly transferable to all locations. Rather, we have explored new conceptual ground and proposed the AVI, with its three critical insights, as a useful heuristic that may assist researchers and practitioners responding to disasters and uncertainty associated with changing social, political and environmental climates at different scales.
